# 51 She Moves - A comparative study examining the perception of the built environment between men and women

**DOI:** 10.1093/eurpub/ckae114.185

**Published:** 2024-09-26

**Authors:** Lauren Duffy

**Affiliations:** Technological University Dublin, Dublin, Ireland

## Abstract

Sustainable urban transport and active travel are part of policy and practice worldwide, with a strong focus on strengthening the relationship between transport and health (US PHA, 2021; NICE, 2012; EU Commission, 2013; ISPAH, 2020). The gap we see in physical activity with males globally being more active than their female counterparts is also evident in transport, with a male bias in planning and design and gender a determinant in modal choice (UN, 2017; ITF, 2018; TII, 2019). This research aims to explore the perceptions of the built environment from a male and female perspective and how this implicates physical activity levels. Walking and cycling for active transport have substantial health benefits, however women are less inclined to use AT than men (Mueller et al, 2015; Garrad et al, 2008; Heesch & Sahlqvist, 2013). This is due to the gendered nature of cites being designed for the use of men and not women (Bassam, 2023).

This study utilised a quantitative approach. A survey was made available nationally (Ireland) on Microsoft Forms, it was available to all genders and both rural and urban individuals. The aim of this survey is gather an in depth understanding of women’s perceptions of the built environment and it’s correlation to activity levels, active travel behaviour and health, and how this differs to men. The Healthy Ireland protocol has been used to gather baseline data, general health and demographic information. The findings have been analysed using SPSS.

The results of this study will be complete before August 2024. The survey is currently available to be completed online. Results are to be published in an academic journal.

The aim of this study is to highlight the difference in perceptions of an individuals built environment dependent on gender. The results from this study will inform policy surrounding the design of cities and towns with infrastructure that is accessible and u​tilized by all. It will inform how we redesign cities for the use of all and have a gender-neutral perspective on city design, active travel and transport.

**Support/Funding Source:**

This research has been funded by Technological University Dublin.

